# A Potential Biofertilizer—Siderophilic Bacteria Isolated From the Rhizosphere of *Paris polyphylla* var. *yunnanensis*

**DOI:** 10.3389/fmicb.2022.870413

**Published:** 2022-05-09

**Authors:** Yihan Wang, Gongyou Zhang, Ya Huang, Min Guo, Juhui Song, Tingting Zhang, Yaohang Long, Bing Wang, Hongmei Liu

**Affiliations:** ^1^Engineering Research Center of Medical Biotechnology, School of Biology and Engineering, Guizhou Medical University, Guiyang, China; ^2^Key Laboratory of Biology and Medical Engineering, Immune Cells and Antibody Engineering Research Center of Guizhou Province, School of Biology and Engineering, Guizhou Medical University, Guiyang, China; ^3^School of Basic Medicine Science, Guizhou Medical University, Guiyang, China

**Keywords:** plant growth promoting rhizobacteria (PGPR), siderophore, iron, *Paris polyphylla* var. *yunnanensis* (*PPVY*), biofertilizers

## Abstract

The increasing demands for crop production have become a great challenge while people also realizing the significance of reductions in synthetic chemical fertilizer use. Plant growth-promoting rhizobacteria (PGPR) are proven biofertilizers for increasing crop yields by promoting plant growth *via* various direct or indirect mechanisms. Siderophilic bacteria, as an important type of PGPR, can secrete siderophores to chelate unusable Fe^3+^ in the soil for plant growth. Siderophilic bacteria have been shown to play vital roles in preventing diseases and enhancing the growth of plants. *Paris polyphylla* var. *yunnanensis* (*PPVY*) is an important traditional Chinese herb. However, reports about its siderophilic bacteria are still rare. This study firstly isolated siderophilic bacteria from the rhizosphere soil of *PPVY*, identified by morphological and physio-biochemical characteristics as well as 16S rRNA sequence analysis. The dominant genus in the rhizobacteria of *PPVY* was *Bacillus*. Among 22 isolates, 21 isolates produced siderophores. The relative amount of siderophores ranged from 4 to 41%. Most of the isolates produced hydroxamate siderophores and some produced catechol. Four isolates belonging to *Enterobacter* produced the catechol type, and none of them produced carboxylate siderophores. Intriguingly, 16 strains could produce substances that have inhibitory activity against *Candida albicans* only in an iron-limited medium (SA medium). The effects of different concentrations of Fe^3+^ and three types of synthetic chemical fertilizers on AS19 growth, siderophore production, and swimming motility were first evaluated from multiple aspects. The study also found that the cell-free supernatant (CFS) with high siderophore units (SUs) of AS19 strain could significantly promote the germination of pepper and maize seeds and the development of the shoots and leaves of *Gynura divaricata* (Linn.). The bacterial solution of AS19 strain could significantly promote the elongation of the roots of *G. divaricata* (Linn.). Due to its combined traits promoting plant growth and seed germination, the AS19 has the potential to become a bioinoculant. This study will broaden the application prospects of the siderophilic bacteria-AS19 as biofertilizers for future sustainable agriculture.

## Introduction

Microorganisms (bacteria and fungi) present in the rhizosphere soil of plants play a significant role in the whole plant growth process (Bukhat et al., 2020). Among these, plant growth-promoting rhizobacteria (PGPR) can promote the plant growth by various direct or indirect mechanisms, such as nitrogen fixation (Raza et al., 2021), phosphate solubilization (Sahu et al., 2022), siderophore production (Braud et al., 2009; Ghazy and El-Nahrawy, 2021), heavy metal resistance (Ma et al., 2016; Bennis et al., 2022), indole compound production (Zahir et al., 2010), biocontrol (Hosseini et al., 2021), and stress responses, including 1-aminocyclopropane-1-carboxylate (ACC) deaminase production (Gupta et al., 2021), drought tolerance (Mansour et al., 2021), and halotolerance (Wang et al., 2022). In the past few decades, many studies on the screening and identification of these bacteria have been reported. Moreover, there is a rich diversity of plant PGPR (Gange and Gadhave, 2018; Yang et al., 2021). Many genera of PGPR have been identified, such as *Bacillus, Brevibacillus, Pseudomonas, Agrobacterium, Burkholderia, Streptomyces*, and *Thiobacillu* (Guo et al., 2020). As reported, the main genera were *norank_c__Cyanobacteria* (53.47%), *Rhizobium* (4.04%), *Flavobacterium* (3.14%), *norank_f__Mitochondria* (2.81%), and *Sphingobium* (2.27%) present in the roots of *PPVY* (Liu et al., 2020).

Iron is an essential element to sustain life activities. It mainly exists in the soil in the form of insoluble ferric Fe(III), and its bioavailability is low (Colombo et al., 2014; Gu et al., 2020). In the environment, Fe(III) forms ferric oxide hydrate complexes (Fe_2_O_3_ × nH_2_O), leading to a free Fe(III) concentration from 10^–9^ to 10^–18^ M. Thus, a plethora of microorganisms, including important human and animal pathogens, are severely restricted in iron acquisition (Miethke and Marahiel, 2007). The uptake of iron by the microorganisms mainly includes direct and indirect mechanisms. Direct mechanisms comprise the uptake of iron sources, including various lactoferrin, transferrin, ferritin, heme, and/or haemoproteins (Wandersman and Delepelaire, 2004). The indirect strategies of iron acquisition are quite diverse. One of these includes exploiting all available iron sources independent of their nature. This makes it the most widespread and successful mechanism of high-affinity iron acquisition in the microbial world (Miethke and Marahiel, 2007). Siderophores are high-affinity ferric ion-specific chelators with a low molecular weight of less than 1.5 kDa that are excreted under iron starvation by various organisms, including bacteria, fungi, and even some plants (Hider and Kong, 2010; Ellermann and Arthur, 2017; Khan et al., 2018). PGPR facilitates plant nutrient uptake from surrounding environments by producing siderophores to sequester iron (Kramer et al., 2020). More than 500 siderophores have been isolated, the majority of which possess either catecholate or hydroxamate functional groups, all of which form Fe(III) complexes with an exceptional stability (Powell et al., 1980).

Plant growth-promoting rhizobacteria that produce siderophores are also called siderophilic bacteria. They are major assets to plants by providing the required amount of iron (Khan et al., 2018). Beneduzi et al. (2012) reported that a potent siderophore plays an important role in the iron uptake by plants in the presence of other metals, such as nickel and cadmium. Gu et al. (2020) indicated that rhizosphere microbiome members with growth-inhibitory siderophores often suppress pathogens *in vitro*, such as in the natural and greenhouse soils (Gu et al., 2020). It has been proven that different kinds of siderophores produced by *Bacillus* spp. play an important role in maintaining the ionic balance and inhibiting the growth of pathogenic microbes (Radhakrishnan et al., 2017). In addition, pathogenic bacteria, such as *Mycobacterium tuberculosis*, continuously chelate their host’s iron by secreting siderophores, causing extensive tissue damage and airway bleeding (Xu et al., 2020). Siderophore-mediated competition for iron drives eco-evolutionary dynamics in natural and infectious settings (Andrews et al., 2003; Cordero et al., 2012).

Siderophores are among the secondary metabolites of PGPR. The rich diversity of secondary metabolites produced by soil bacteria has been recognized for over a century. Screening of microbial extracts reveals the high structural diversity of natural compounds with broad biological activities, such as antimicrobial (Zhanel et al., 2019; Chakraborty et al., 2022), antiviral (Jakubiec-Krzesniak et al., 2018), immunosuppressive (Duan et al., 2020), pesticide (Keswani et al., 2020), biodeterioration (David et al., 2021), and antitumor activities (Wang and Ding, 2018), enabling the bacteria to survive in its natural environment. The lipopeptide produced by *Bacillus amyloliquefaciens* YN201732, mainly bacillomycin D, showed great biocontrol activity against the fungal pathogen, *Fusarium solani* (Jiao et al., 2021). Therefore, it is of great value and significance to study siderophilic bacteria and their secondary metabolite activity.

*Paris polyphylla* var. *yunnanensis (PPVY)* is one of the most important traditional Chinese herbs. The rhizomes of *PPVY* have become important medicinal materials due to their extensive pharmacological activities, including hemostatic, detoxification, detumescence, analgesic, immune regulating, and antibacterial, anti-inflammatory, and antitumor functions (Yun et al., 2007; Chan et al., 2011; Negi et al., 2014; Qin et al., 2016; Guo et al., 2018). However, the siderophilic bacteria in the rhizosphere of *PPVY* have not been studied systematically. Here, we report the diversity of siderophilic bacteria isolated from the rhizosphere of *PPVY* and the types and relative amounts of siderophores produced by the siderophilic bacteria. We found that the AS19 strain has the potential to be used as a biofertilizer. In addition, the antimicrobial activity of secondary metabolites of siderophilic bacteria was screened, and the influence of synthetic chemical fertilizers on siderophilic bacterial growth and siderophore production was investigated. Seed germination and plant growth promotion experiments were also carried out. These data provide a basis for the study of efficient bioinoculants and the development of green agriculture.

## Materials and Methods

### Rhizosphere Soil Sampling

Samples of rhizosphere soil of *P. polyphylla* var. *yunnanensis* were collected from Yanla township (105.968°E, 26.041°N) Xi Xiu district, An shun city, Guizhou Province, China. The excess soil was first gently shaken from the roots, and the remaining soil attached to the tubers of *P. polyphylla var. yunnanensis* was considered the rhizosphere soil.

### Isolation of Rhizobacteria

To isolate bacteria, 10 g of rhizosphere soil was mixed with 90 mL of sterile distilled water in a rotary shaker at 180 rpm for 30 min at 30°C. After serial dilution in sterile distilled water, 100 μl volumes of the diluted soil suspensions were plated on Luria–Bertani (LB) medium plates (peptone 10 g/L, yeast extract 5 g/L, sodium chloride 10 g/L, and agar 1.5 g/L). After a 24-h incubation at 37°C, colonies with clear morphological characteristics were selected, purified, and preserved in liquid LB medium. To avoid potential fungal contamination, only highly diluted samples were used for isolation. The isolates were then restreaked on LB plates for colony purification. The final collection consisted of 22 bacterial isolates from rhizosphere soil samples. All purified isolates were cultured in 5 ml of LB medium at 37°C with shaking (rotary shaker at 180 rpm) for 12 h before being frozen and stored at −80°C with 15% glycerol.

### Identification of Rhizobacteria

#### Morphology

The purified bacteria were streaked on LB agar medium and incubated at 37°C for 12 h. The morphology, color, edge, and the shape of the bacterial colonies were observed with reference to Bergey’s Manual of Systematic Bacteriology (Whitman, 2015). Moreover, Gram staining was examined with reference to Hans Christian Gram’s method conducted in 1884.

#### Physiology and Biochemistry

Starch hydrolysis, gelatine liquefaction, sugar fermentation, methyl red (MR), indole, Voges- Proskauer reaction, citrate, and hydrogen sulfide tests were performed to determine the physiological and biochemical characteristics of the pure strains.

#### 16S Ribosomal RNA Gene Sequencing and Phylogenetic Analysis of Rhizobacteria

Total genomic DNA was extracted from overnight cultures of the individual bacterial isolates, which were cultured in LB medium at 37°C with shaking (200 rpm.), using the Bacterial Genomic DNA Extraction Kit (TianGen CAT# dp302-02) according to the manufacturer’s protocol. The 16S rRNA gene was amplified by PCR using the universal primers, 27F (5′-AGAGTTTGATCMTGGCTCAG-3′) and 1492R (5′-TACGGTTACCTTGTTACGACTT-3′). The PCR amplification was carried out on A600 Super Gradient (LongGene) in a mixture (50 μL) containing 10 × PCR buffer (5 μL), DNA (2 μL), dNTPs (4 μL), primer (1 μL), Taq polymerase (0.5 μL, TaKaRa), and double distilled water (36.5 μL) with 30 cycles as follows: denaturation at 94°C for 1 min, annealing at 56°C for 1 min, and elongation at 72°C for 1.5 min. Predenaturation was carried out at 95°C for 5 min, and final elongation was performed at 72°C for 10 min. The PCR products were detected by agarose gel electrophoresis and sequenced (supported by Sangon Biotech, Shanghai, China).

The 16S rRNA sequences of 22 isolates were analysed for alignment with sequences in the NCBI database using the Blastn method.^1^ The phylogenetic trees were constructed using the neighbor-joining (NJ) algorithm with MEGA 7.0 (Kumar et al., 2018). The strengths of the internal branches of the resulting trees were statistically evaluated by bootstrap analysis with 1,000 bootstrap replications.

### Detection of Siderophore Production in Rhizobacteria

To quantify the siderophore production, the isolates were inoculated into LB medium. One hundred microliters of bacteria culture was spread on an SA-CAS plate (1/2 SA medium + 1/2 CAS medium) and incubated at 30°C for 48 h. Siderophore production was analysed using a modified version of the universal chemical assay developed by Schwyn and Neilands (1987) and Silva-Bailao et al. (2014).

The liquid CAS assay was used to quantify the concentrations of siderophores. Specifically, we harvested the cell-free supernatant (CFS) from 1 mL of SA bacterial cultures by centrifugation (12,000 rpm, 10 min at 4°C) and filtration (using a 0.22 μm filter). Then, 100 μl of CFS (three biological replicates for all 22 soil isolates) and SA medium as a control reference were added to 100 μl of CAS assay solution in a 96-well plate. After 0.5–1 h of static incubation at room temperature, the OD_630_ of the CFSs (As) and SA medium controls (Ar) were then measured using a microplate reader (Elx800 microplate reader, BioTek) at room temperature. Siderophores induce a color change in the CAS medium, which lowers the OD_630_ measurements, and siderophore production can be quantified using the following formula (Guo et al., 2016):


$$\mathrm{Siderophore}\mathrm{Unit}\left(\mathrm{SU}\right)=\frac{\mathrm{Ar}-\mathrm{As}}{\mathrm{Ar}}\times 100\%$$

Ar–the absorbance of the reference (SA medium) at 630 nm. As–the absorbance of the sample (CFS/bacterial cultures) at 630 nm.

### Determining the Type of Siderophores Produced by Rhizobacteria

Each isolate was cultured in 15 mL of LB medium at 37°C and 200 rpm for 12 h. Then, 1.5 mL of bacterial solution was inoculated into 50 mL of SA medium. Cells were grown for 48 h at 30°C and 200 rpm. Then, 1 mL of bacterial culture was centrifuged at 12,000 rpm for 10 min to obtain CFS for subsequent experiments.

#### Hydroxamates

The *FeCl_3_ test* (Schwyn and Neilands, 1987) was used to detect hydroxamate-type siderophores. Then, 0.5–1 mL of 2% FeCl_3_ solution was added to 0.5 mL of SA fermentation supernatants. The formation of red or purple colors indicated the presence of hydroxamate siderophores. An absorption peak between 420–450 nm indicated a hydroxamate nature. Peaks were noted on a UV–visible spectrophotometer (2802 UV/VIS SPECTROPHOTOMETER, UNICO).

#### Catecholates

*Arnow’s* test (Arnow, 1937; Sheng et al., 2020) was used to detect catecholate-type siderophores. First, 0.5 mL of SA fermentation supernatant was added to 0.5 mL of 0.5 M HCl and 0.5 mL of reagent containing 10 g each of NaNO_2_ and Na_2_MoO_4_⋅2H_2_O in 100 ml water. When 0.5 mL of 1 M NaOH was added, the color of the solution turned from yellow to red. Absorbance was measured at 515 nm to indicate the presence of catecholic siderophores (2802 UV/VIS PECTROPHOTOMETER, UNICO).

#### Carboxylates

*Shenker’s test* (Shenker et al., 2008) was used to detect carboxylate-type siderophores. First, 0.5 mL of 250 μM CuSO_4_ and 1 mL of acetate buffer (pH = 4) were added to 0.5 mL of SA fermentation filtrate. The copper complex formed was observed between 190 and 280 nm to determine the maximum absorption. There is no specific wavelength at which the copper complex is absorbed. The entire wavelength from 190–280 nm was scanned to observe the peaks of siderophore absorption (2802 UV/VIS PECTROPHOTOMETER, UNICO).

### Antimicrobial Activity of Rhizobacteria

To determine whether the CFS of SA or LB culture fermentation for 22 rhizobacteria isolates had an antimicrobial effect, gram-positive *Staphylococcus aureus*, gram-negative *Escherichia coli*, and the fungus *Candida albicans* were selected as indicator microbes. In this study, the agar diffusion method (the punch method) was used to investigate the antimicrobial activity of the siderophore solution. Each rhizobacterial isolate was inoculated in SA and LB medium at 30°C and 200 rpm for 48 h, respectively. The CFS of bacterial culture from SA and LB medium were prepared by centrifugation (12,000 rpm, 10 min at 4°C) and filtration (using a 0.22 μm filter). *Escherichia coli* and *Staphylococcus aureus* were cultured on LB agar medium at 37°C for 12–14 h. *Candida albicans* was cultured on PDA at 30°C for 14–16 h. Wells approximately 8 mm in diameter and 2 mm deep were made on the surface of the agar medium using a sterile borer. Each well was subsequently filled with 100 μL of test sample and labeled with a marker. The same amount of sterile SA medium was used as a negative control, while amphotericin and cephalosporin were used as positive controls. The size of the bacteriostatic zone was observed after overnight culture.

### Effect of Different Concentrations of Iron on the Growth and Siderophore Production of the AS19 Strain

FeCl_3_ was added to 150 mL of SA medium at a final concentration of 0, 100, 200, 400, 600, 800, 1,000, and 2,000 μM Fe^3+^, 3–5 ml of bacterial culture was collected every 6 h. The bacterial growth was quantified spectrophotometrically by measuring the optical density at 600 nm. One milliliter of bacterial culture was centrifuged at 12,000 rpm for 10 min at the indicated time points. As described above, the CFS was detected by CAS assay and the siderophores units (SUs) were calculated quantitatively to evaluate the effects of different iron concentrations on the growth and the synthesis of siderophores of AS19 strain.

### Evaluation of the Antimicrobial Activity of AS19 at Different Fe^3+^ Concentrations

AS19 strain was inoculated in 150 mL of SA medium with final concentrations of 0, 100, 200, 400, 600, 800, 1,000, and 2,000 μM Fe^3+^ and cultured at 30°C for 48 h. One milliliter of the bacterial culture was collected and centrifuged at 12,000 rpm for 10 min. The antimicrobial experiment was performed as described above.

### Effects of Urea, KH_2_PO_4_, and Nitrogen, Phosphorus, and Potassium on the Growth and Siderophore Production of AS19

Urea, KH_2_PO_4_ was added to 150 mL of SA medium, and the final concentration was 10, 50, 90 mg/mL. Nitrogen, phosphorus, and potassium (NPK) synthetic chemical fertilizers (N:P:K = 15:15:15) was also added to 150 mL of SA medium, and the final concentration was 5, 20, 30, and 50 mg/mL. AS19 bacterial broth was inoculated in SA medium containing different concentration of urea, KH_2_PO_4_ and NPK at 30°C, 200 rpm. Bacterial growth (OD_600_) and SUs were measured at some point in time. The calculation of the siderophore units was performed as described above.

### Effects of Different Concentrations of Fe^3+^ on the Motility of AS19

The swimming motility of AS19 strain was analysed using solid LB medium with 0.3% of agar by adding different concentrations of Fe^3+^, following a method published previously with some modifications (Chen et al., 2012; Gao et al., 2020). The addition of 100 μM 2,2-bipyridine was performed to establish to an iron-free environment. A single colony of AS19 was inoculated in 0.3% of agar medium and cultured at 30°C for swarming assays. Motility diameter was measured 16–24 h after inoculation.

### Assessment of Growth-Promoting Traits *in vitro*

#### Indole-3-Acetic Acid Production

Indole-3-acetic acid (IAA) production by the isolate *Bacillus altitudinis* strain AS19 was determined according to the methodology described by Bric et al. (1991). The amount of IAA produced by AS19 was determined in a multimode reader (Cytation 5, BioTek) at 530 nm. The concentration of IAA produced by the bacterial isolate AS19 was calculated from the standard curve of commercial IAA (Sigma, United States) in the range of 10–100 μg/mL.

#### Qualitative Estimation of Ammonia Production

Ammonia production by the bacterial isolate AS19 was determined by following the methodology of Cappuccino and Sherman (1992).

#### Solubilization of Phosphate

The phosphate solubilization ability of the bacterial isolate AS19 was determined in Pikovskaya agar medium according to Pikovskaya, R.I.’s method (Pikovskaya, 1948) with the solubilization index calculated as follows:


$$\begin{array}{c} \mathrm{Solubilization}\mathrm{Index}\left(\mathrm{SI}\right)\\ =\frac{\mathrm{Colony}\mathrm{diameter}\left(\mathrm{mm}\right)+\mathrm{Halozone}\mathrm{diameter}\left(\mathrm{mm}\right)}{\mathrm{Colony}\mathrm{diameter}\left(\mathrm{mm}\right)} \end{array}$$

#### Organic Acid Production

AS19 was spot inoculated on M9 agar medium with methyl red as a pH indicator dye. Plates were incubated for three days at 28 ± 2°C (Sambrook and Russell, 2001).

#### Antagonism Experiment

Soilborne pathogenic fungi and bacteria including *Fusarium oxysporum*, *Fusarium solanum*, and *Pectobacterium carotovorum* were as indicator microbes to observe the inhibition zone in antagonism experiments.

### Seed Germination Assay

Hot pepper and maize seeds were purchased at random and soaked in a dishwashing liquid, washed in running water for 30 min, and disinfected with 75% alcohol for 30 s. Then, the seeds were washed with sterile water 3–5 times, sterilized with 3% of sodium hypochlorite for 10 min, and rewashed with sterile water 4–5 times. The seeds were placed on presterilized filter paper in separate Petri plates (three replicates). First, 20 ml of CFS, bacterial solution, and SA medium were applied to each group. Seeds were kept damp by spraying 5 ml of the above active components on filter paper every 5 days. Plates with the hot pepper and maize seeds were incubated at 25 ± 1°C, and any physiological changes in each group of seeds were recorded. The experiment was carried out in a completely randomized design under controlled conditions. The experiment was performed in triplicate.

### Evaluation of Plant Growth Promotion by AS19

*Gynura divaricata* (Linn.) was used to verify whether strain AS19 can promote plant growth. The seedlings were cultured in MS medium (with 0.02 mg/L NAA, 3% sucrose, 0.7% agar) for 15–18 days, and then experiments were carried out. The inoculated seedlings with uniform growth were selected, and the sealing film was removed to allow the seedlings to adapt to the external environment for 3 days. Then, the terminal buds, lateral leaves, and lateral roots were cut off. The remaining 5–6 primary roots were kept in the same initial state as the seedlings. The seedlings were transplanted into the same sized sterilized pot (9.5 cm high, 9 cm diameter) with a mixture of sterile sand and soil in the ratio of 3:1. The disinfection of the pots was carried out using 75% ethanol and washed with sterile water several times to remove chemicals. The soil was mixed thoroughly and then added to the pots for the control and treated seedlings. This step ensured that all the plants obtained the same nutrients. After transplantation, the plants were irrigated with 100 ml of water for the first time. After 5 days, the plants were randomly divided into three groups: the supernatant group, bacterial solution group, and SA medium group, with five pots in each group. Different nutrients of the experimental group and control group were added to the plants regularly, 20 ml each time, once every 4 days. Day and night were simulated by incandescent light. The temperature was maintained at 25 ± 3°C. After 40 days, the growth parameters of the plants were recorded and compared with those of the control plants. The parameters considered were the number of leaves, number of shoots, shoot length, root length, and stem length.

### Toxicity Test of *Hermetia illucens* L. Larvae

The CFS of AS19 was lyophilized and dissolved with saline. *Hermetia illucens* L. larvae were fed wheat bran and housed in an environmentally controlled breeding room (temperature: 28–30°C; humidity: 60 ± 5%, 12 h dark/light cycle). The *H. illucens* L. larvae were divided into 5 groups (*n* = 50 larvae/group). The natural growth group was only punctured. The control group was injected with 2.5 μL of sterile saline. The experimental group was injected with 2.5 μL of CFS (30 mg/kg, 40 mg/kg, 1 g/kg). The survival rate and changes in body weight were observed and recorded every 24 h for 7 days.

### Reproducibility of the Results and Statistical Analysis

The assays were repeated independently three times. In each assay, the values obtained at each time point were further measured in triplicate. The results are presented as an average of the total determination. The standard deviations associated with each average value were represented in the form of error bars. The mean values were further subjected to one-way ANOVA followed by the Tukey–Kramer multiple comparison method; *P*-values less than 0.05 were considered statistically significant.

### Nucleotide Sequence Accession Numbers

The sequences of the 16S rRNA gene of the 22 isolates identified in this study have been deposited in the National Center for Biotechnology Information (NCBI) Nucleotide database. The accession numbers were shown in Supplementary Table 1.

## Results

### Isolation and Identification the Rhizobacteria of *Paris polyphylla* var. *yunnanensis*

In our study, 22 strains of bacteria were isolated and used in the following experiments.

#### Morphological Characteristics

**FIGURE 1:**
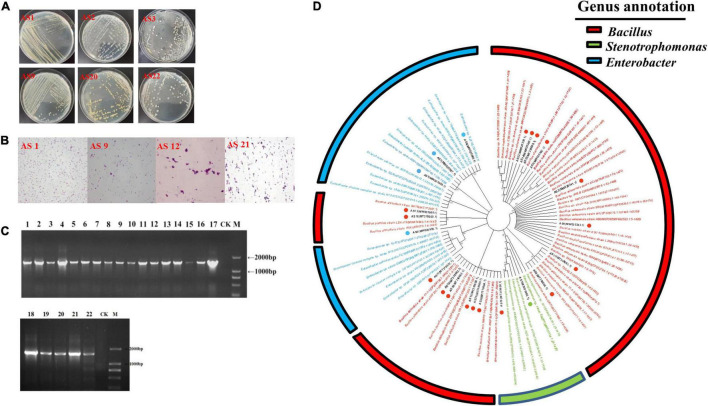
Screening and identification of rhizosphere bacteria. **(A)** Representative colony morphology. After repeated isolation, monoclones were selected and cultured in LB liquid medium for 12–14 h at 37°C. The purified strain was streaked onto LB agar medium by the streak plate method, and the colony morphology was observed. **(B)** Gram staining. AS1 and AS9 represent gram-negative bacteria, and AS12 and AS21 represent gram-positive bacteria. **(C)** PCR results for 16S rRNA sequencing amplification of the 22 bacterial isolates. M: DL2000 DNA marker, CK: blank, the numbers 1–17 represent the bacterial isolates AS1, AS2, AS3, AS4, AS5, AS6, AS7, AS8, AS9, AS10, AS11, AS12, AS13, AS14, AS15, AS16, and AS17, and the numbers 18–22 represent the bacterial isolates AS18, AS19, AS20, AS21, and AS22, respectively. **(D)** Phylogenetic trees of the 22 bacterial isolates based on their 16S rRNA sequences.

Morphologically distinct bacterial isolates were selected and studied for their colony characteristics. The size, shape, edge, transparency, and color of the isolates are described in Supplementary Table 2. The plates are shown in Figure 1A. Some isolates had similar morphological characteristics, such as AS3 and AS22. Gram staining results showed that AS1, AS5, AS6, AS7, and AS9 were gram-negative bacteria, while the others were gram-positive (Figure 1B).

#### Physiological and Biochemical Characteristics

The physiological and biochemical characteristics of these 22 strains are shown in Supplementary Table 3. Although AS3 and AS22 had similar morphological characteristics, the results of starch hydrolysis, gelatine liquefaction, citrate, and hydrogen sulfide tests of AS22 were positive, while those of AS3 were negative. Similarly, although AS5, AS6, and AS7 had similar morphological characteristics, the Voges–Proskauer reaction of AS6 showed positive results, which was different from AS5 and AS7.

#### Molecular Identification

The PCR products were observed as a single band with a size of approximately 1,500 bp (Figure 1C). The NJ tree based on 16S rRNA sequences showed that the 22 isolates were grouped into 3 different genera: *Bacillus*, *Enterobacter*, and *Stenotrophomonas* (Figure 1D). The *Bacillus* group contained 17 isolates, while the small group belonging to the *Stenotrophomonas* genus included only one isolate. The results suggested that the dominant genus in the rhizosphere of *PPVY* was *Bacillus*. Interestingly, 70.59% (12/17) of the isolates belonged to the *Bacillus pumilus* group but had different physiological and biochemical characteristics.

### Siderophore Synthesis Validated in Rhizobacteria

**FIGURE 2:**
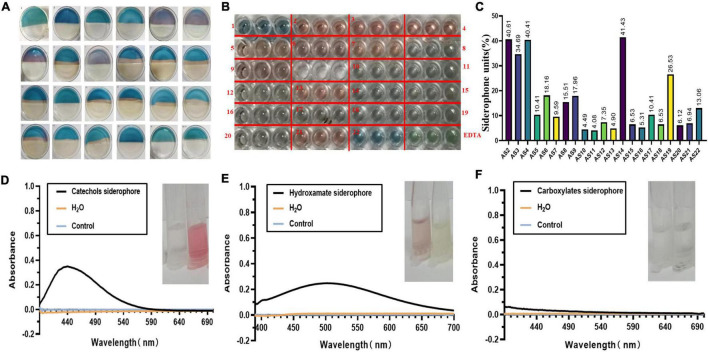
The units and type of siderophores produced by 22 isolates. **(A)** SA-CAS agar plate assay. Isolates were cultured on SA-CAS agar plates, and the blue color changed to pink or yellow. **(B)** CAS liquid testing was performed in a 96-well plate. **(C)** The units of siderophores produced by 21 isolates calculated according to SU = (Ar − As)/Ar*100%. The absorbance was measured at 630 nm. **(D)** Catecholate-type siderophore. AS2, AS3, AS4, AS5, AS6, AS7, AS9, AS14, and AS22 absorption peaks were detected at 440 nm. **(E)** Hydroxamate-type siderophore. The absorption peaks of AS8, AS10, AS11, AS12, AS13, AS15, AS16, AS17, AS18, AS19, AS20, and AS21 were detected at 505 nm. **(F)** Carboxylate-type siderophore. No absorption peak was detected in any of the samples at 190–280 nm.

Combined with CAS plate and liquid assays, we found that 21 isolates could secrete siderophores. Among them, nine isolates named AS2, AS3, AS4, AS5, AS6, AS7, AS9, AS13, and AS21 turned the blue color of the agar to pink in CAS liquid assays (Figure 2A). Another 12 isolates, including AS8, AS10, AS11, AS12, AS14, AS15, AS16, AS17, AS18, AS19, AS20, and AS22, produced a color change from blue to yellow (Figure 2A).

### Different Types of Siderophores Produced by Rhizobacteria

We found that the SUs of each isolate were between 4 and 41% (Figures 2B,C). The maximum amount of siderophore production, 41.40%, was observed from the AS14 isolate. With the Arnow method, when NaOH was added to the SA fermentation supernatant, the AS2, AS3, AS4, AS5, AS6, AS7, AS9, AS14, and AS22 solutions immediately turned red, and absorption peaks were also detected at 440 nm by full wavelength scanning. The siderophore produced by these nine strains was identified as the catecholate type (Figure 2D). The hydroxamate type was identified by the FeCl_3_ method. When 2% of FeCl_3_ was added, the solutions of AS8, AS10, AS11, AS12, AS13, AS15, AS16, AS17, AS18, AS19, AS20, and AS21 turned red. After full wavelength scanning, absorption peaks were detected at 505 nm (Figure 2E). The siderophores produced by these 12 strains were identified as the hydroxamate type. In Shenker’s method, the maximum absorption peak of the sample was not detected at 190–280 nm. The carboxylic type of siderophore was not found in this study (Figure 2F).

### Antimicrobial Activity of Rhizobacteria

**FIGURE 3:**
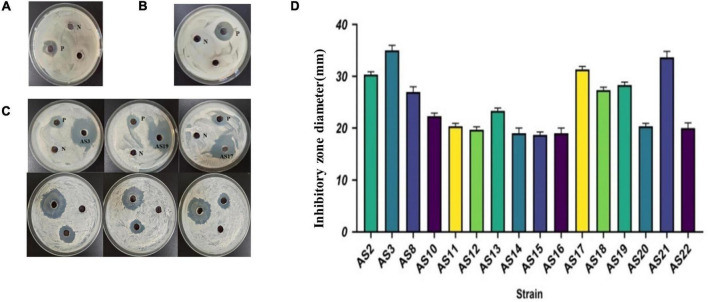
The antimicrobial activity of CFSs of 22 isolates grown on SA. **(A)** Antimicrobial experiment with SA CFS against *Escherichia coli* N: SA medium negative control, P: Cephalosporin positive control. **(B)** Antimicrobial experiment with SA CFS against *Staphylococcus aureus.* N: SA medium negative control, P: cephalosporin positive control. **(C)** Antimicrobial experiment with SA CFS against *C. albicans.* N: SA medium negative control, P: Amphotericin positive control. **(D)** The inhibitory zone of 16 strains against *C. albicans*. Represented by individual plate experiments, the data statistics are shown in Table 1.

Among the 22 rhizobacteria isolated from *PPVY*, only the SA medium CFS of 16 strains, AS2, AS3, AS8, AS10, AS11, AS12, AS13, AS14, AS15, AS16, AS17, AS18, AS19, AS20, AS21, and AS22, could significantly inhibit the growth of *Candida albicans* (Table 1 and Figure 3C). The inhibition zones of AS3, AS17, and AS21 were larger than those of the positive control (3 mg/ml, amphotericin). The inhibition zone diameters are shown in Figure 3D. However, no inhibition zone was observed near the sample wells on the *Staphylococcus aureus* and *Escherichia coli* plates (Figures 3A,B). In addition, the CFS of LB medium did not show antimicrobial activity against any of the three indicator microbes (Table 1 and Supplementary Figure 1). These results demonstrated that the metabolites of 16 strains of siderophilic bacteria contain active substances that have a strong inhibitory effect on fungi. SA medium is a low-iron medium and is often used to induce siderophore production. Based on this, we speculated that the antimicrobial substance may be siderophore related.

**TABLE 1:** *In vitro* antimicrobial activity of cell-free supernatant (CFS) of bacterial strains.

Indicator bacteria	*Staphylococcus aureus*		*Escherichia coli*		*Candida albicans*	
Cell-free supernatant	LB	SA	LB	SA	LB	SA
AS1						
AS2						**++**
AS3						**+++**
AS4						–
AS5						–
AS6						–
AS7						–
AS8						**++**
AS9						–
AS10						**+**
AS11						**+**
	–	–	–	–	–	
AS12						**+**
AS13						**++**
AS14						**+**
AS15						**+**
AS16						**+**
AS17						**+++**
AS18						**++**
AS19						**++**
AS20						**+**
AS21						**+++**
AS22						**+**

*−, no inhibition; +, inhibition;++, clear inhibition; +++, very clear inhibition.*

### Effect of Different Concentrations of Fe^3+^ on AS19 Siderophore Production

**FIGURE 4:**
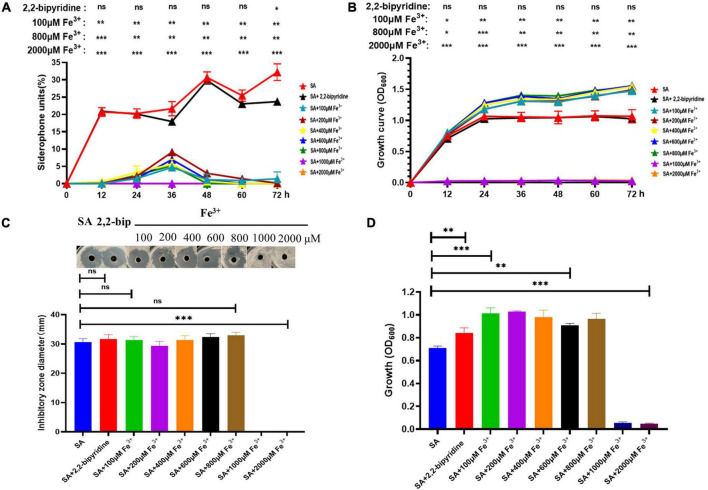
Growth curve, siderophore production, and bacteriostasis of AS19 at different Fe^3+^ concentrations. **(A)** Effect of different concentrations of Fe^3+^ on AS19 siderophore production. AS19 was cultured in SA medium containing different concentrations of Fe^3+^ at 30°C and 200 rpm. **(B)** Effect of different concentrations of Fe^3+^ on AS19 growth. AS19 was inoculated into SA medium containing different concentrations of Fe^3+^ at 30°C and 200 rpm. **(C)** Effect of different concentrations of Fe^3+^ on AS19 bacteriostatic activity. AS19 was inoculated into SA medium containing different concentrations of Fe^3+^ at 30°C and 200 rpm for 48 h. One milliliters of bacterial solution were centrifuged at 12,000 rpm for 10 min. The CFS was used for the plate bacteriostasis test. **(D)** Effect of different concentrations of Fe^3+^ on AS19 growth at 48 h. (ns, no significant difference, *p < 0.05, ^**^p < 0.01, ^***^p < 0.001).

The AS19 strain was chosen for further study since it shows the largest inhibition zone and higher siderophore production units. Studies have shown that siderophore production is inhibited in the presence of excess iron. SA medium containing different concentrations of Fe^3+^ was used to determine the siderophore units produced by AS19 in different time periods. As shown in Figure 4B, the presence of an appropriate amount of Fe^3+^ could promote the growth of AS19, but when the Fe^3+^ concentration was more than 1,000 μM, AS19 stopped growing. When the concentration of Fe^3+^ was more than 100 μM, AS19 produced very little or no siderophores (Figure 4A), suggesting that the addition of Fe^3+^ inhibited the secretion of siderophores.

### Antimicrobial Activity of AS19 Cell-Free Supernatant at Different Fe^3+^ Concentrations

To explore the effect of Fe^3+^ on the antimicrobial activity of AS19, CFS after 48 h of fermentation in SA medium with different concentrations of Fe^3+^ was utilized. The results showed that when the Fe^3+^ concentration was over 1,000 μM, the AS19 strain no longer grew, and the antimicrobial activity of the CFS was also lost (Figures 4C,D). As soon as the concentration of Fe^3+^ was over 100 μM, the siderophore production of AS19 was inhibited, but its antimicrobial activity was not affected. SA medium with different concentrations of Fe^3+^ was used as a negative control, and no inhibition zone was produced (Supplementary Figure 2). Therefore, we suspected that there were no siderophores produced by AS19 but other secondary metabolites or a combination of various substances that exerted antimicrobial activity on *Candida albicans*.

### Effects of Synthetic Chemical Fertilizers on AS19 Growth and Siderophore Production

**FIGURE 5:**
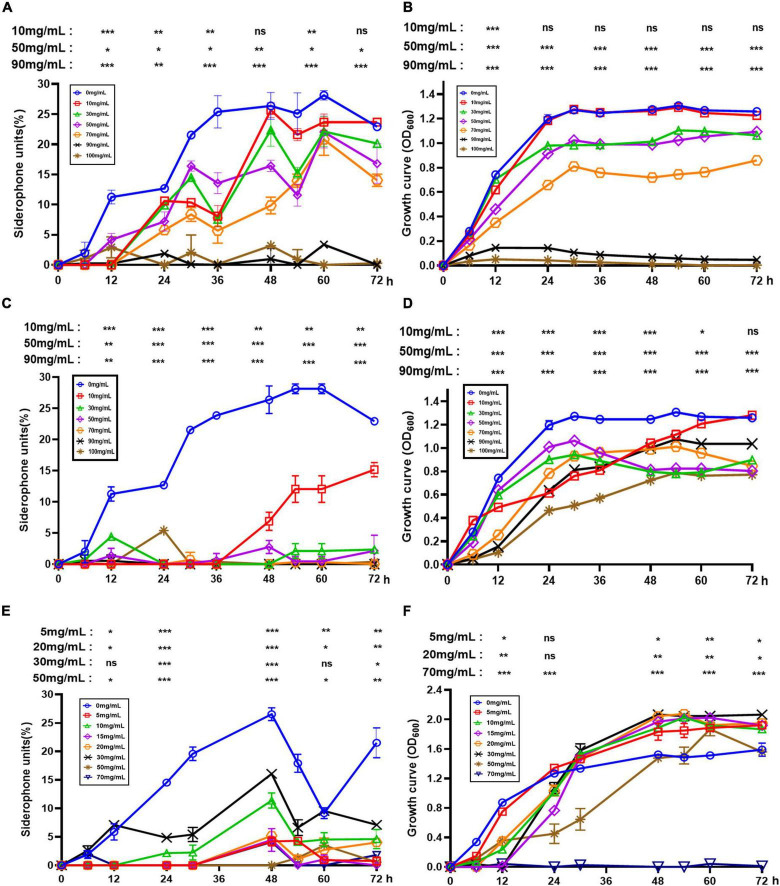
Effects of urea, KH_2_PO_4_ and NPK on the growth and siderophore production of AS19. **(A)** Siderophore production at different concentrations of urea. **(B)** The growth curve at different concentrations of urea. **(C)** Siderophore production at different concentrations of KH_2_PO_4_. **(D)** The growth curve at different concentrations of KH_2_PO_4_. **(E)** Siderophore production at different concentrations of NPK compound fertilizer. **(F)** The growth curve at different concentrations of NPK compound fertilizer (ns, no significant difference, *p < 0.05, **p < 0.01, ***p < 0.001).

To further investigate the effect of fertilizers frequently used in farming on siderophore synthesis in AS19, a series of experiments were performed with urea, KH_2_PO_4_, and NPK fertilizers. As shown in Figure 5B, with increasing urea concentration, the growth of AS19 was inhibited. When the urea concentration was above 90 g/L, AS19 basically stopped growing. Similarly, a high concentration of KH_2_PO_4_ inhibited the growth of AS19 (Figure 5D). At the same time, when the concentration was 100 g/L, the biomass of AS19 could only reach half of that under normal conditions. The growth of AS19 was first inhibited and then promoted by the addition of NPK fertilizer. An appropriate amount of NPK fertilizer (≤30 g/L) was conducive to the growth of AS19, but excessive use of NPK fertilizer (≥50 g/L) could inhibit or even stop the growth of AS19 (Figure 5F). Overall, there was a phenomenon of promotion at low concentrations and inhibition at high concentrations.

On this basis, we detected the synthesis of siderophores by AS19. The addition of urea and NPK fertilizer inhibited the synthesis of siderophores overall (Figures 5A,E). The addition of KH_2_PO_4_ caused AS19 to produce siderophores no longer in the case of normal growth (Figure 5C). In conclusion, excess urea and KH_2_PO_4_ were not conducive to the growth and siderophore production of the AS19 strain. At low concentrations, NPK fertilizer slightly promoted the growth of AS19 but inhibited the secretion of siderophores. Therefore, the use of various synthetic chemical fertilizers limited the role of AS19 as a PGPR.

### Effects of Different Concentrations of Fe^3+^ on the Swimming Motility of AS19

**FIGURE 6:**
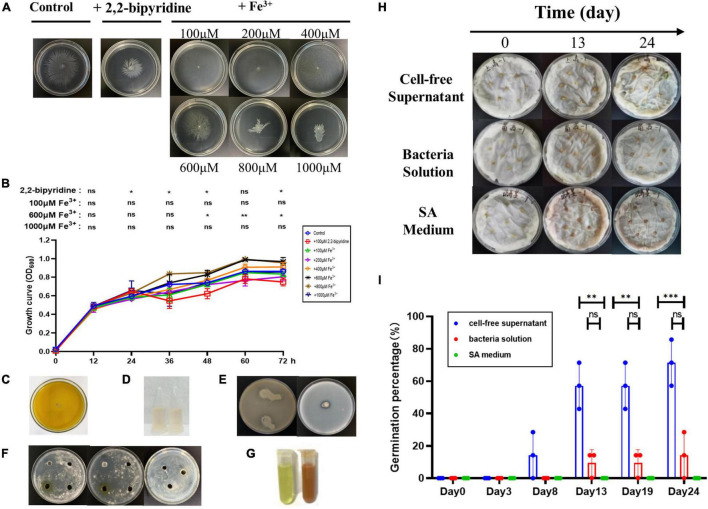
Swimming motility, growth-promoting and seed-germination assays. **(A)** The swimming of AS19 in solid LB medium with 0.3% agar under different concentrations of Fe^3+^. **(B)** Effect of different concentrations of Fe^3+^ on the growth of AS19. **(C)** Production of organic acids. **(D)** Production of IAA. **(E)** Decomposition of phosphate. **(F)** Antagonism experiment. **(G)** Production of ammonia. **(H)** The germination of pepper seeds treated with CFS and bacterial solution of AS19. The SA medium group was the control group. **(I)** The germination percentage of pepper seeds (a total of seven in each group). The experiment was repeated three times. Three dots represent the germination percentage of three experiments (ns, no significant difference, *p < 0.05, ^**^p < 0.01, ^***^p < 0.001).

Motility is one of the most important traits for efficient rhizosphere colonization. In this study, the growth of AS19 was not affected by different concentrations of Fe^3+^, but the swimming ability showed a different trend (Figures 6A,B). When the concentration was between 100 and 400 μM, the motility was strong and could extend to the edge of the plate. As the concentration reached 600 μM or above, the swimming ability declined, and the motility diameter decreased. Overall, low concentrations of iron promoted the swimming motility of the AS19 strain, while high concentrations inhibited swimming motility.

### Plant Growth Promoting and Antagonism Traits of AS19

The AS19 isolates showed negative results for organic acid (Figure 6C) and IAA production (Figure 6D). In phosphate solubilization, a slightly transparent circle appeared on the inorganic phosphorus plate, while this was not observed in organic phosphorus, suggesting that it had a slight effect on phosphate solubilization (Figure 6E). The sample turned red to indicate ammonia production, proving that AS19 could produce ammonia (Figure 6G). Furthermore, AS19 can produce the hydroxamate type of siderophore, which is an important bioactive substance. Thus, AS19 could be considered a PGPR to some extent. However, AS19 could not antagonize *F. oxysporum*, *F. solanum*, or *P. carotovorum* in the antagonism assays (Figure 6F).

### Effects of AS19 on Seed Germination

To further understand the biofertilization potential of AS19, the effect on germination was observed and recorded. The final germination rate of pepper seed in CFS group was stable at 71% (5/7), which was nearly 10 times higher than that of the bacterial solution group (7.1%), but no seeds germinated in the medium group (Figure 6I). Furthermore, we found that pepper seeds in the supernatant group began to germinate on the 8th day, and seeds in the solution group only began to germinate on the 13th day (Figure 6H). Similarly, the CFS also promoted the germination of maize seeds, and the germination rate was up to 85.71% (6/7), which was six times higher than the SA medium group (Figures 8B,C). Our experiments indicated that the secondary metabolites of AS19 could promote the germination of pepper and maize seeds.

### Plant Growth Promotion of *Gynura divaricata* (Linn.) by AS19

**FIGURE 7:**
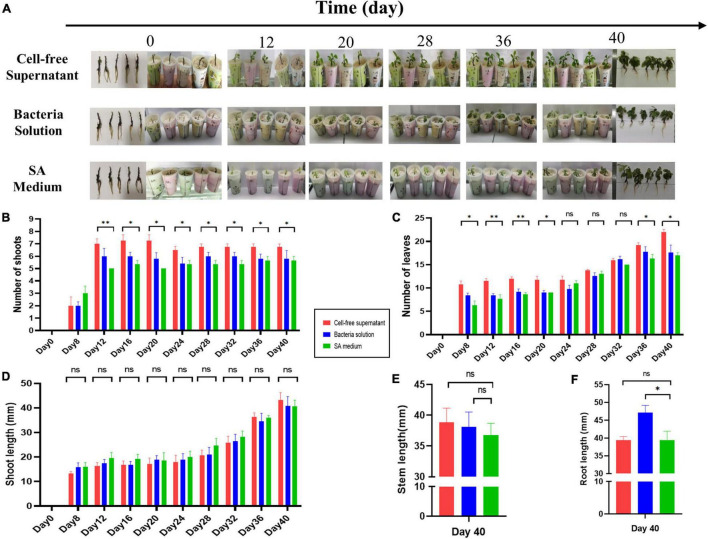
Plant growth promoting effects of AS19. **(A)** Evaluation of the growth parameters of *Gynura divaricata* (*Linn.*) treated with CFS and bacterial solution of AS19. The SA medium group was the control group. **(B)** Number of shoots. **(C)** Number of leaves. **(D)** Shoot length. **(E)** Stem length. **(F)** Root length (ns, no significant difference, *p < 0.05, ^**^p < 0.01).

The growth parameters of the SA medium group and CFS and bacterized *Gynura divaricata* (Linn.) seedlings were compared, and the results are presented in Figure 7A. Treatment with AS19 CFS increased the germination number and leaf number of *Gynura divaricata* (Linn.) plants by up to 1.45 times (Figure 7B) and 1.57 times (Figure 7C), respectively, compared to the control group. Root elongation was significantly promoted by the bacterial solution by 1.19 times (Figure 7F). However, there was no significant difference in the length of germination and stems, and the supernatant group and the bacterial solution group had slightly higher values than the SA medium group (Figures 7D,E).

### Prediction of Gene Clusters for Siderophore Synthesis

**FIGURE 8:**
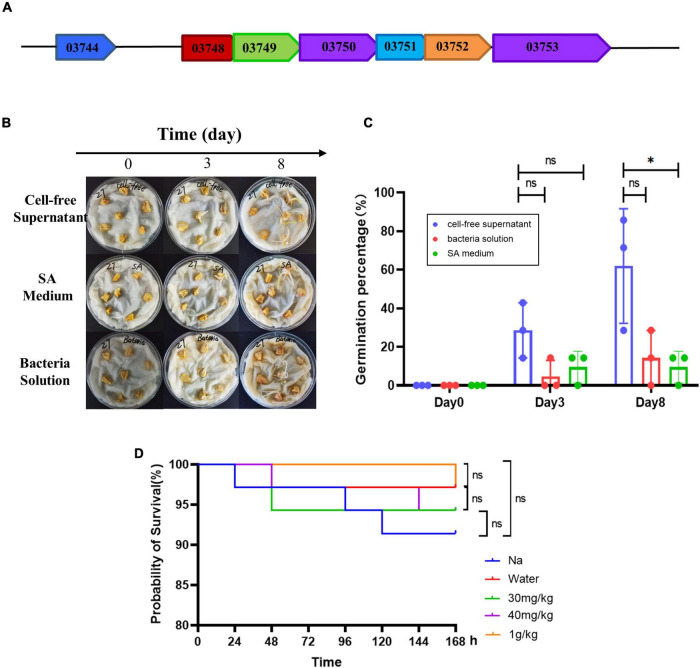
Gene prediction, maize germination, and toxicity assessment. **(A)** Prediction of genes related to siderophore synthesis in AS19. The arrow direction indicates the direction of transcription. The overlapped of arrows indicates the base sequence of overlap. **(B)** The germination of maize seeds treated with CFS and the bacterial solution of AS19. The SA medium group was the control group. **(C)** The germination percentage of maize seeds (a total of seven in each group). The experiment was repeated three times. Three dots represent the germination percentage of three experiments (ns, no significant difference, *p < 0.05). **(D)** Toxicity test of *Hermetia illucens* L. larvae. Improved survival rate of *H. illucens* L. larvae with CFS. *H. illucens* L. larvae were randomly divided into five groups. The natural growth (NG) group was only punctured. The control (Control) group was injected with 2.5 μL of sterile saline. The experimental group was injected with 2.5 μL of CFS (30 mg/kg, 40 mg/kg, and 1 g/kg). The number of surviving larvae was observed every 24 h, for 7 days. The survival rates were calculated, and the graph was drawn with GraphPad Prism 8.4 software (ns, no significant difference, *p < 0.05, *n* = 50).

The siderophore synthesis genes of AS19 strain was preliminarily analysed and the pattern was mapped (Figure 8A). The siderophore synthesis gene cluster, including six core synthesis genes (*AS19_03748, AS19_03749, AS19_03750, AS19_03751, AS19_03752*, and *AS19_03753*). The analysis by the software, Hmmscan showed that the main genes of siderophore synthesis were *AS19_03750* and *AS19_03753*. One transcriptional regulatory gene *AS19_03744* located at the upstream of the siderophore synthesis gene cluster, was similar to *AlcR* in *Bordetella pertussis* (Accession no. AF018255.1), *AraC* in *Escherichia coli* (Accession no. V00259.1), and this transcriptional activator could positively regulate the production and transport of siderophore. It was also found that there were short base overlaps among six siderophore synthesis genes (Figure 8A).

### Evaluation of the Biosafety of AS19 as a Biofertilizer

In order to evaluate the biosafety of AS19 as a biological fertilizer, *H. illucens* L. larvae was used as a test subject. The results of survival curve showed that the dosage of 30 mg/mL, 40 mg/mL, and 1 g/kg had no effect on the survival of *H. illucens* L. larvae (Figure 8D). These results implicated the safety of the CFS of AS19 fermentation liquid in organisms.

## Discussion

Siderophilic bacteria, a kind of plant growth-promoting bacteria, have attracted much attention and been investigated for decades. These bacteria can promote plant growth and regulate the soil microenvironment by secreting siderophores (Powell et al., 1980; Ellermann and Arthur, 2017; Khan et al., 2018; Kramer et al., 2020). In our study, 22 strains were isolated from the rhizosphere soil of *P. polyphylla* var. *yunnanensis* and divided into three groups, including the species of *Bacillus, Enterobacter*, and *Stenotrophomonas* (Figure 1D). The small group belonging to the *Stenotrophomonas* genus contained only one isolate. The dominant genus, *Bacillus*, contained 17 isolates. Twelve isolates with different physio-biochemical characteristics were identified as the *B. pumilus* group by 16S rRNA gene sequence analysis. The *B. pumilus* group contains five species, *B. pumilus*, Bacillus *safensis*, *Bacillus stratosphericus*, *Bacillus altitudinis*, and *Bacillus aerophilus*. Apart from the AS1 strain belonging to *Stenotrophomonas*, 95.45% (21/22) of isolates could secrete siderophores (Figure 2B). These results showed that siderophilic bacteria were abundant in the rhizosphere soil of *PPVY*. The *B. pumilus* group corresponded to the dominant siderophilic bacteria.

Studies have indicated that rhizosphere microorganisms of plants secrete one or more siderophores to prevent plant diseases (Zhou et al., 2019). To evaluate the siderophore production of 22 isolates, a CAS assay was performed. The AS14 strain showed the highest production of siderophores, which was 41.40% (Figure 2C). Two types of siderophores, catecholates and hydroxamates, were found in our experiment. We also found that *Enterobacter* mainly produced catecholic-type siderophores, which is consistent with the report of Neilands (1981). Habibi et al. (2019) reported that *Pseudomonas* species were more active in terms of siderophore production than species of other genera. It is interesting to note that no *Pseudomonas* isolate was identified in our study. The different results may have been due to the bacterial species varying with the rhizospheres of different plants and different locations.

The secondary metabolites produced by these isolated strains in our work had a significant inhibitory effect on *C. albicans* but there was no inhibitory effect on G**^–^** *E. coli* and G**^+^** *S. aureus* (Figure 3). When the 16 strains were cultured in LB or SA media, we found that only the fermentation filtrate in the low-iron SA medium showed strong antimicrobial activity against *C. albicans* (Table 1). It has been reported that siderophores are widely used not only in plant pest control (Hosseini et al., 2021), heavy metal pollution remediation (Yi et al., 2022), and fertilizer supplementation (Orr et al., 2020) but also in combination with antifungal drugs for the initial treatment of fungal infections, such as *Cryptococcus* (Chayakulkeeree et al., 2020; Sheng et al., 2020). The hydroxamate siderophore desferrioxamine B has been included in the World Health Organization (WHO) demonstration list of essential drugs for the treatment of iron load in patients with thalassaemia (Aydinok et al., 2015). These results implied that the siderophores secreted by the 16 isolated strains have antimicrobial activity against *C. albicans*.

To further explore the effect of Fe^3+^ on siderophores, the *B. altitudinis* strain AS19, which had the best bacteriostatic activity and high siderophore units, was selected. The presence of a high concentration of Fe^3+^ significantly inhibited the growth of AS19. Concentrations exceeding 1,000 μM Fe^3+^ were toxic to the bacteria. However, the presence of 100 μM Fe^3+^ greatly reduced the production of siderophores, which could not be detected under these conditions. To investigate whether the antifungal activity of AS19 was related to the production of siderophores, we investigated the influence of the presence of Fe^3+^ on its antimicrobial activity. The results showed that the presence of Fe^3+^ at low concentrations did not affect the inhibition of *C. albicans* (Figure 4C). We speculated that the bacteriostatic activity might not mainly be caused by the siderophore or that there were other active components, such as bacteriocin, non-ribosomal peptides, dihydro-iso-coumarins, cyclic lipodepsipeptides, and linear lipodepsipeptides with antimicrobial properties in the metabolites of *Bacillus*, which played a bacteriostatic role together with the siderophore (Wang and Ding, 2018; Kaspar et al., 2019).

To further explore the biofertilizer potential of AS19, the effects of different chemical fertilizers on the growth and siderophore production of AS19 were creatively assessed. Urea and K_2_HPO_4_ significantly inhibited the growth of AS19 and reduced its siderophore production. Although the low concentration of NPK fertilizer had the tendency to promote the growth of AS19, it still inhibited the production of siderophores. This is consistent with the results of recent reports on the soil toxicity of chemical fertilizers and the destruction of the microbial ecological environment (Rahman et al., 2020). Long-term intensive fertilization may increase the risk of soil degradation due to the sensational modifications in soil microbial diversity (Sun et al., 2019; Tang et al., 2021). In addition, excessive use of chemical fertilizer harms the soil environment, leading to problems, such as soil acidification (Barak et al., 1997; Liu et al., 2010), soil compaction (Pagliai et al., 2004), and NP runoff loss (Baffaut et al., 2019). Cumulatively, these problems can easily lead to water eutrophication and the destruction of the steady state of affected aquatic ecosystems (Cui et al., 2018). Soil microorganisms have limited tolerance to fertilizers. Fertilizers could affect the growth and the traits of PGPR (Figure 5). Studies have shown that an appropriate reduction in fertilization is beneficial to achieve a win–win situation of environmental and economic benefits (Wang et al., 2020). Thus, it is necessary to use chemical fertilizers as little as possible in agricultural plantings. Moreover, an increasing number of biofertilizers and new potential PGPRs need to be utilized in future agriculture.

Swimming motility is important for PGPR distribution in soil. Interestingly, we found that a low concentration of Fe^3+^ promoted the swimming ability of the AS19 strain, while excessive Fe^3+^ inhibited its swimming ability (Figure 6A). It has not been reported that genes related to swimming motility in *Bacillus* are regulated by Fe^3+^. As a siderophore bacteria isolated from the rhizosphere of *PPVY*, the properties of AS19 as a plant-growth promoter in the rhizosphere of plants was explored. It was found that AS19 could produce siderophores, ammonia, and phosphorus (Figure 6G) but not inhibit the growth of the soil pathogens *F. oxysporum*, *F. solanum*, and *P. carotovorum* (Figure 6F). Further, seed germination and plant growth promotion experiments showed that the CFS with high SUs and a variety of bioactive substances in its metabolites, could promote the germination of pepper and maize seeds (Figures 6H,I), and the development of the shoots and leaves of *G. divaricata* (Linn.) (Figures 7B,C). Chloroplasts are important organs for buds and leaves, and iron ions are important for chloroplasts. The CFS of AS19, with high SUs, could promote the transport of unavailable Fe^3+^ in the environment into plant cells. It was speculated that the shoots and leaves developed well because of the abundance of siderophore and nutrients in AS19 fermentation CFS. The reason why the bacterial solution could significantly promote the root elongation (Figure 7F) was that the siderophilic bacteria, AS19 had good motility (Figure 6) and could colonize in the roots of *G. divaricata* (Linn.) to promote elongation.

The siderophore synthesis gene clusters of AS19 were preliminarily predicted (Figure 8A), *AS19_03750* and *AS19_03753* as the two main synthetic gene, and *AS19_03744* as the upstream transcriptional regulation factor should be further studied. In addition, the germination experiment of maize also confirmed the growth promoting effect of CFS with 26.53% of the SUs (Figures 8B,C). The biotoxicity of *H. illucens* L. larvae was evaluated as having no toxicity (Figure 8D). Furthermore, even more species need to be involved in biosafety assessment, but the existing results indicate the growth-promoting role of siderophore and the potential application prospects of siderophilic bacteria, AS19 as a biofertilizer.

**FIGURE 9:**
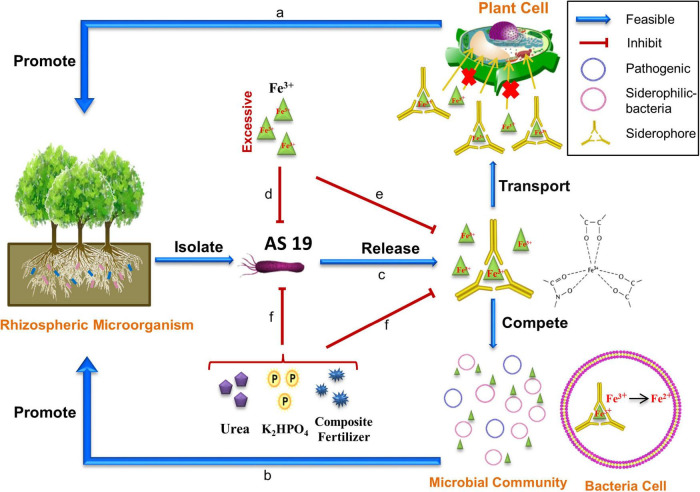
Schematic of the possible plant growth promoting pathway by AS19. **(a)** Fe^3+^ cannot enter plant cells directly, but the complex is transported across the membrane into the plant to help it absorb nutrients. **(b)** Siderophores alter the microbial community. Pathogenic microbes do not grow due to a lack of bioavailable iron. **(c)** AS19 secretes siderophores. **(d)** In LB medium with 0.3% agar, the addition of iron had no effect on the growth of AS19, but a low concentration of Fe^3+^ promoted motility, and excessive Fe^3+^ inhibited motility. **(e)** In a low-iron environment, excessive Fe^3+^ significantly inhibited the growth of AS19 and the production of siderophores. **(f)** In a low-iron environment, chemical fertilizer inhibited the growth of AS19 and the secretion of siderophores to varying degrees. Interestingly, the addition of low-dose compound fertilizer slightly increased the bacterial growth but still inhibited the siderophore production.

In general, the role of AS19 as a growth promoter might be mediated by siderophores (Figure 9). The AS19 facilitates the absorption of Fe^3+^ by seeds and plants or competes with pathogenic microorganisms for iron resources through the secretion of siderophores. Different types of siderophores promote the growth of several plant species and increase their yield by enhancing Fe uptake by plants. The activities of siderophores as iron chelators have been shown in different studies, such as siderophores from *Chryseobacterium* spp. C138, which were effective in the supply of iron in tomato plants when delivered to the roots (Saha et al., 2016). Another case was seen in the supplementation of *Pseudomonas* strains, which showed a significant increase in germination and plant growth (Sharma and Johri, 2003).

The present investigation isolated and identified 22 strains of rhizosphere bacteria of *PPVY* and analysed the abundance of siderophilic bacteria. The metabolites of 16 strains with inhibitory effects on *C. albicans* were screened. Meanwhile, the effects of Fe^3+^ and different fertilizers on PGPR AS19 were further explored. This study establishes the promoting effect of the AS19 strain on seed germination and plant growth as a PGPR. This study paves the way for the further discovery of new compounds of anti-*Candida albicans* and the development of environmentally friendly biofertilizers, PGPR, for future sustainable agricultural development.

## Data Availability Statement

The datasets presented in this study can be found in online repositories. The names of the repository/repositories and accession number(s) can be found in the article/Supplementary Material.

## Author Contributions

YW contributed to the design and implementation of this study as well as preparation and drafting of the manuscript. GZ, YH, JS, MG, TZ, and YL coordinated and participated in the data analysis and contributed to the discussion of the manuscript. BW and HL contributed to the design and implementation of the research work and revised the manuscript. All authors analysed and discussed the data and reviewed and approved the manuscript.

## Conflict of Interest

The authors declare that the research was conducted in the absence of any commercial or financial relationships that could be construed as a potential conflict of interest.

## Publisher’s Note

All claims expressed in this article are solely those of the authors and do not necessarily represent those of their affiliated organizations, or those of the publisher, the editors and the reviewers. Any product that may be evaluated in this article, or claim that may be made by its manufacturer, is not guaranteed or endorsed by the publisher.

## Footnotes

1
https://blast.ncbi.nlm.nih.gov/Blast.cgi

## References

[B1] Andrews S. C. Robinson A. K. Rodríguez-Quiñones F. (2003). Bacterial iron homeostasis. *FEMS Microbiol. Rev.* 27 215–237. 10.1016/s0168-6445(03)00055-x 12829269

[B2] Arnow L. E. (1937). Colorimetric determination of the components of 3,4-dihydroxyphenylalanine-tyrosine mixtures. *J. Biol. Chem.* 118 531–537. 10.1016/s0021-9258(18)74509-2

[B3] Aydinok Y. Kattamis A. Cappellini M. D. El-Beshlawy A. Origa R. Elalfy M. (2015). Effects of deferasirox-deferoxamine on myocardial and liver iron in patients with severe transfusional iron overload. *Blood* 125 3868–3877. 10.1182/blood-2014-07-586677 25934475 PMC4490296

[B4] Baffaut C. Ghidey F. Lerch R. N. Kitchen N. R. Sudduth K. A. Sadler E. J. (2019). Long-term simulated runoff and water quality from grain cropping systems on restrictive layer soils. *Agric. Water Manag.* 213 36–48. 10.1016/j.agwat.2018.09.032

[B5] Barak P. Jobe B. O. Krueger A. R. Peterson L. A. Laird D. A. (1997). Effects of long-term soil acidification due to nitrogen fertilizer inputs in Wisconsin. *Plant Soil* 197 61–69.

[B6] Beneduzi A. Ambrosini A. Passaglia L. M. P. (2012). Plant growth-promoting rhizobacteria (PGPR): their potential as antagonists and biocontrol agents. *Genet. Mol. Biol.* 35 1044–1051. 10.1590/s1415-47572012000600020 23411488 PMC3571425

[B7] Bennis M. Perez-Tapia V. Alami S. Bouhnik O. Lamin H. Abdelmoumen H. (2022). Characterization of plant growth-promoting bacteria isolated from the rhizosphere of *Robinia pseudoacacia* growing in metal-contaminated mine tailings in eastern Morocco. *J. Environ. Manage.* 304:114321. 10.1016/j.jenvman.2021.114321 35021593

[B8] Braud A. Jézéquel K. Bazot S. Lebeau T. (2009). Enhanced phytoextraction of an agricultural Cr- and Pb-contaminated soil by bioaugmentation with siderophore-producing bacteria. *Chemosphere* 74 280–286. 10.1016/j.chemosphere.2008.09.013 18945474

[B9] Bric J. M. Bostock R. M. Silverstone S. E. (1991). Rapid in situ assay for indoleacetic acid production by bacteria immobilized on a nitrocellulose membrane. *Appl. Environ. Microbiol.* 57 535–538. 10.1128/aem.57.2.535-538.1991 16348419 PMC182744

[B10] Bukhat S. Imran A. Javaid S. Shahid M. Majeed A. Naqqash T. (2020). Communication of plants with microbial world: exploring the regulatory networks for PGPR mediated defense signaling. *Microbiol. Res.* 238:126486. 10.1016/j.micres.2020.126486 32464574

[B11] Cappuccino J. G. Sherman N. (1992). *Microbiology: a Laboratory Manual.* New York, NY: Pearson.

[B12] Chakraborty K. Kizhakkekalam V. K. Joy M. Chakraborty R. D. (2022). Bacillibactin class of siderophore antibiotics from a marine symbiotic *Bacillus* as promising antibacterial agents. *Appl. Microbiol. Biotechnol.* 106 329–340. 10.1007/s00253-021-11632-0 34913995

[B13] Chan J. Y. Koon J. C. Liu X. Detmar M. Yu B. Kong S. K. (2011). Polyphyllin D, a steroidal saponin from *Paris polyphylla*, inhibits endothelial cell functions in vitro and angiogenesis in zebrafish embryos in vivo. *J. Ethnopharmacol.* 137 64–69. 10.1016/j.jep.2011.04.021 21658438

[B14] Chayakulkeeree M. Tangkoskul T. Waywa D. Tiengrim S. Pati N. Thamlikitkul V. (2020). Impact of iron chelators on growth and expression of iron-related genes of *Cryptococcus* species. *J. Mycol. Med.* 30:100905. 10.1016/j.mycmed.2019.100905 31706700

[B15] Chen Y. Chai Y. Guo J. H. Losick R. (2012). Evidence for cyclic Di-GMP-mediated signaling in *Bacillus subtilis*. *J. Bacteriol.* 194 5080–5090. 10.1128/JB.01092-12 22821967 PMC3430322

[B16] Colombo C. Palumbo G. He J.-Z. Pinton R. Cesco S. (2014). Review on iron availability in soil: interaction of Fe minerals, plants, and microbes. *J. Soils Sediments* 14 538–548. 10.1007/s11368-013-0814-z

[B17] Cordero O. X. Ventouras L. A. Delong E. F. Polz M. F. (2012). Public good dynamics drive evolution of iron acquisition strategies in natural bacterioplankton populations. *Proc. Natl. Acad. Sci. U.S.A.* 109 20059–20064. 10.1073/pnas.1213344109 23169633 PMC3523850

[B18] Cui Z. Zhang H. Chen X. Zhang C. Ma W. Huang C. (2018). Pursuing sustainable productivity with millions of smallholder farmers. *Nature* 555 363–366. 10.1038/nature25785 29513654

[B19] David S. R. Jaouen A. Ihiawakrim D. Geoffroy V. A. (2021). Biodeterioration of asbestos cement by siderophore-producing *Pseudomonas*. *J. Hazard. Mater.* 403:123699. 10.1016/j.jhazmat.2020.123699 32853889

[B20] Duan X. Tan X. Gu L. Liu J. Hao X. Tao L. (2020). New secondary metabolites with immunosuppressive activity from the phytopathogenic fungus *Bipolaris maydis*. *Bioorg. Chem.* 99:103816. 10.1016/j.bioorg.2020.103816 32305693

[B21] Ellermann M. Arthur J. C. (2017). Siderophore-mediated iron acquisition and modulation of host-bacterial interactions. *Free Radic. Biol. Med.* 105 68–78. 10.1016/j.freeradbiomed.2016.10.489 27780750 PMC5401654

[B22] Gange A. C. Gadhave K. R. (2018). Plant growth-promoting rhizobacteria promote plant size inequality. *Sci. Rep.* 8:13828. 10.1038/s41598-018-32111-z 30218023 PMC6138649

[B23] Gao T. Ding M. Wang Q. (2020). The recA gene is crucial to mediate colonization of *Bacillus cereus* 905 on wheat roots. *Appl. Microbiol. Biotechnol.* 104 9251–9265. 10.1007/s00253-020-10915-2 32970180

[B24] Ghazy N. El-Nahrawy S. (2021). Siderophore production by *Bacillus subtilis* MF497446 and *Pseudomonas koreensis* MG209738 and their efficacy in controlling *Cephalosporium maydis* in maize plant. *Arch. Microbiol.* 203 1195–1209. 10.1007/s00203-020-02113-5 33231747 PMC7683328

[B25] Gu S. Wei Z. Shao Z. Friman V. P. Cao K. Yang T. (2020). Competition for iron drives phytopathogen control by natural rhizosphere microbiomes. *Nat. Microbiol.* 5 1002–1010. 10.1038/s41564-020-0719-8 32393858 PMC7116525

[B26] Guo H. Yang Y. Liu K. Xu W. Gao J. Duan H. (2016). Comparative genomic analysis of *Delftia tsuruhatensis* MTQ3 and the identification of functional NRPS genes for siderophore production. *Biomed Res. Int.* 2016:3687619. 10.1155/2016/3687619 27847812 PMC5099486

[B27] Guo Y. Liu Z. Li K. Cao G. Sun C. Cheng G. (2018). *Paris Polyphylla*-derived saponins inhibit growth of bladder cancer cells by inducing mutant P53 degradation while up-regulating CDKN1A expression. *Curr. Urol.* 11 131–138. 10.1159/000447207 29692692 PMC5903466

[B28] Guo Y. Ren C. Yi J. Doughty R. Zhao F. (2020). Contrasting responses of rhizosphere bacteria, fungi and arbuscular mycorrhizal fungi along an elevational gradient in a temperate montane forest of China. *Front. Microbiol.* 11:2042. 10.3389/fmicb.2020.02042 32973736 PMC7469537

[B29] Gupta A. Bano A. Rai S. Kumar M. Ali J. Sharma S. (2021). ACC deaminase producing plant growth promoting rhizobacteria enhance salinity stress tolerance in *Pisum sativum*. *3 Biotech* 11:514. 10.1007/s13205-021-03047-5 34926112 PMC8630178

[B30] Habibi S. Djedidi S. Ohkama-Ohtsu N. Sarhadi W. A. Kojima K. Rallos R. V. (2019). Isolation and screening of indigenous plant growth-promoting rhizobacteria from different rice cultivars in afghanistan soils. *Microbes Environ.* 34 347–355. 10.1264/jsme2.ME18168 31527341 PMC6934389

[B31] Hider R. C. Kong X. (2010). Chemistry and biology of siderophores. *Nat. Prod. Rep.* 27 637–657. 10.1039/b906679a 20376388

[B32] Hosseini A. Hosseini M. Schausberger P. (2021). Plant growth-promoting rhizobacteria enhance defense of strawberry plants against spider mites. *Front. Plant Sci.* 12:783578. 10.3389/fpls.2021.783578 35069641 PMC8770953

[B33] Jakubiec-Krzesniak K. Rajnisz-Mateusiak A. Guspiel A. Ziemska J. Solecka J. (2018). Secondary metabolites of actinomycetes and their antibacterial, antifungal and antiviral properties. *Pol. J. Microbiol.* 67 259–272. 10.21307/pjm-2018-048 30451442 PMC7256786

[B34] Jiao R. Cai Y. He P. Munir S. Li X. Wu Y. (2021). *Bacillus amyloliquefaciens* YN201732 produces lipopeptides with promising biocontrol activity against fungal pathogen *Erysiphe cichoracearum*. *Front. Cell. Infect. Microbiol.* 11:598999. 10.3389/fcimb.2021.598999 34222035 PMC8253258

[B35] Kaspar F. Neubauer P. Gimpel M. (2019). Bioactive secondary metabolites from *Bacillus subtilis*: a comprehensive review. *J. Nat. Prod.* 82 2038–2053. 10.1021/acs.jnatprod.9b00110 31287310

[B36] Keswani C. Singh H. B. Garcia-Estrada C. Caradus J. He Y. W. Mezaache-Aichour S. (2020). Antimicrobial secondary metabolites from agriculturally important bacteria as next-generation pesticides. *Appl. Microbiol. Biotechnol.* 104 1013–1034. 10.1007/s00253-019-10300-8 31858191

[B37] Khan A. Singh P. Srivastava A. (2018). Synthesis, nature and utility of universal iron chelator - Siderophore: a review. *Microbiol. Res.* 212-213 103–111. 10.1016/j.micres.2017.10.012 29103733

[B38] Kramer J. Ozkaya O. Kummerli R. (2020). Bacterial siderophores in community and host interactions. *Nat. Rev. Microbiol.* 18 152–163. 10.1038/s41579-019-0284-4 31748738 PMC7116523

[B39] Kumar S. Stecher G. Li M. Knyaz C. Tamura K. (2018). MEGA X: molecular evolutionary genetics analysis across computing platforms. *Mol. Biol. Evol.* 35 1547–1549. 10.1093/molbev/msy096 29722887 PMC5967553

[B40] Liu E. Yan C. Mei X. He W. Bing S. H. Ding L. (2010). Long-term effect of chemical fertilizer, straw, and manure on soil chemical and biological properties in northwest China. *Geoderma* 158 173–180. 10.1016/j.geoderma.2010.04.029

[B41] Liu T. H. Zhou Y. Tao W. C. Liu Y. Zhang X. M. Tian S. Z. (2020). Bacterial diversity in roots, stems, and leaves of chinese medicinal plant *Paris polyphylla* var. *yunnanensis*. *Pol. J. Microbiol.* 69 91–97. 10.33073/pjm-2020-012 32189484 PMC7256839

[B42] Ma Y. Oliveira R. S. Freitas H. Zhang C. (2016). Biochemical and molecular mechanisms of plant-microbe-metal interactions: relevance for phytoremediation. *Front. Plant Sci.* 7:918. 10.3389/fpls.2016.00918 27446148 PMC4917562

[B43] Mansour E. Mahgoub H. A. M. Mahgoub S. A. El-Sobky E. E. A. Abdul-Hamid M. I. Kamara M. M. (2021). Enhancement of drought tolerance in diverse *Vicia faba* cultivars by inoculation with plant growth-promoting rhizobacteria under newly reclaimed soil conditions. *Sci. Rep.* 11:24142. 10.1038/s41598-021-02847-2 34921154 PMC8683512

[B44] Miethke M. Marahiel M. A. (2007). Siderophore-based iron acquisition and pathogen control. *Microbiol. Mol. Biol. Rev.* 71 413–451. 10.1128/MMBR.00012-07 17804665 PMC2168645

[B45] Negi J. S. Bisht V. K. Bhandari A. K. Bhatt V. P. Singh P. Singh N. (2014). *Paris polyphylla*: chemical and biological prospectives. *Anticancer Agents Med. Chem.* 14 833–839. 10.2174/1871520614666140611101040 24917072

[B46] Neilands J. B. (1981). Microbial iron compounds. *Ann. Rev. Biochem.* 50 715–731. 10.1146/annurev.bi.50.070181.003435 6455965

[B47] Orr R. Hocking R. K. Pattison A. Nelson P. N. (2020). Extraction of metals from mildly acidic tropical soils: interactions between chelating ligand, pH and soil type. *Chemosphere* 248:126060. 10.1016/j.chemosphere.2020.126060 32032879

[B48] Pagliai M. Vignozzi N. Pellegrini S. (2004). Soil structure and the effect of management practices. *Soil Tillage Res.* 79 131–143. 10.1016/j.still.2004.07.002

[B49] Pikovskaya R. I. (1948). Mobilization of phosphorus in soil connection with the vital activity of some microbial species. *Mikrobiologiya* 17 362–370.

[B50] Powell P. E. Cline G. R. Rcidt C. P. P. Szaniszlo P. J. (1980). Occurrence of hydroxamate siderophore iron chelators in soils. *Nature* 287 833–834. 10.1038/287833a0

[B51] Qin X. J. Yu M. Y. Ni W. Yan H. Chen C. X. Cheng Y. C. (2016). Steroidal saponins from stems and leaves of *Paris polyphylla* var. *yunnanensis*. *Phytochemistry* 121 20–29. 10.1016/j.phytochem.2015.10.008 26546502

[B52] Radhakrishnan R. Hashem A. Abd Allah E. F. (2017). *Bacillus*: a biological tool for crop improvement through bio-molecular changes in adverse environments. *Front. Physiol.* 8:667. 10.3389/fphys.2017.00667 28932199 PMC5592640

[B53] Rahman M. M. Nahar K. Ali M. M. Sultana N. Karim M. M. Adhikari U. K. (2020). Effect of long-term pesticides and chemical fertilizers application on the microbial community specifically anammox and denitrifying bacteria in rice field soil of jhenaidah and kushtia district, Bangladesh. *Bull. Environ. Contam. Toxicol.* 104 828–833. 10.1007/s00128-020-02870-5 32385520

[B54] Raza A. Ejaz S. Saleem M. S. Hejnak V. Ahmad F. Ahmed M. A. A. (2021). Plant growth promoting rhizobacteria improve growth and yield related attributes of chili under low nitrogen availability. *PLoS One* 16:e0261468. 10.1371/journal.pone.0261468 34919599 PMC8683023

[B55] Saha M. Sarkar S. Sarkar B. Sharma B. K. Bhattacharjee S. Tribedi P. (2016). Microbial siderophores and their potential applications: a review. *Environ. Sci. Pollut. Res. Int.* 23 3984–3999. 10.1007/s11356-015-4294-0 25758420

[B56] Sahu S. Rajbonshi M. P. Gujre N. Gupta M. K. Shelke R. G. Ghose A. (2022). Bacterial strains found in the soils of a municipal solid waste dumping site facilitated phosphate solubilization along with cadmium remediation. *Chemosphere* 287:132320. 10.1016/j.chemosphere.2021.132320 34826951

[B57] Sambrook J. Russell D. (2001). *Molecular Cloning: A Laboratory Manual*, 3rd Edn. Cold Spring Harbor, NY: Cold Spring Harbor Laboratory Press.

[B58] Schwyn B. Neilands J. B. (1987). Universal chemical assay for the detection and determination of siderophores. *Anal. Biochem.* 160 47–56. 10.1016/0003-2697(87)90612-9 2952030

[B59] Sharma A. Johri B. N. (2003). Growth promoting influence of siderophore-producing *Pseudomonas* strains GRP3A and PRS9 in maize (*Zea mays* L.) under iron limiting conditions. *Microbiol. Res.* 158 243–248. 10.1078/0944-5013-00197 14521234

[B60] Sheng M. M. Jia H. K. Zhang G. Y. Zeng L. N. Zhang T. T. Long Y. H. (2020). Siderophore production by rhizosphere biological control bacteria *Brevibacillus brevis* GZDF3 of pinellia ternata and its antifungal effects on *Candida albicans*. *J. Microbiol. Biotechnol.* 30 689–699. 10.4014/jmb.1910.10066 32482934 PMC9728291

[B61] Shenker M. Oliver I. Helmann M. Hadar Y. Chen Y. (2008). Utilization by tomatoes of iron mediated by a siderophore produced byRhizopus arrhizus. *J. Plant Nutr.* 15 2173–2182. 10.1080/01904169209364466

[B62] Silva-Bailao M. G. Bailao E. F. Lechner B. E. Gauthier G. M. Lindner H. Bailao A. M. (2014). Hydroxamate production as a high affinity iron acquisition mechanism in *Paracoccidioides* spp. *PLoS One* 9:e105805. 10.1371/journal.pone.0105805 25157575 PMC4144954

[B63] Sun R. Li W. Hu C. Liu B. (2019). Long-term urea fertilization alters the composition and increases the abundance of soil ureolytic bacterial communities in an upland soil. *FEMS Microbiol. Ecol.* 95:fiz044. 10.1093/femsec/fiz044 30947327

[B64] Tang H. Li C. Shi L. Xiao X. Cheng K. Wen L. (2021). Effect of different long-term fertilizer managements on soil nitrogen fixing bacteria community in a double-cropping rice paddy field of southern China. *PLoS One* 16:e0256754. 10.1371/journal.pone.0256754 34469461 PMC8409621

[B65] Wandersman C. Delepelaire P. (2004). Bacterial iron sources: from siderophores to hemophores. *Annu. Rev. Microbiol.* 58 611–647. 10.1146/annurev.micro.58.030603.123811 15487950

[B66] Wang G. Li B. Peng D. Zhao H. Lu M. Zhang L. (2022). Combined application of H2S and a plant growth promoting strain JIL321 regulates photosynthetic efficacy, soil enzyme activity and growth-promotion in rice under salt stress. *Microbiol. Res.* 256:126943. 10.1016/j.micres.2021.126943 34953293

[B67] Wang K. W. Ding P. (2018). New bioactive metabolites from the marine-derived fungi aspergillus. *Mini Rev. Med. Chem.* 18 1072–1094. 10.2174/1389557518666180305160856 29512458

[B68] Wang Z. Geng Y. Liang T. (2020). Optimization of reduced chemical fertilizer use in tea gardens based on the assessment of related environmental and economic benefits. *Sci. Total Environ.* 713:136439. 10.1016/j.scitotenv.2019.136439 31954250

[B69] Whitman W. B. (2015). *Bergey’s Manual of Systematics of Archaea and Bacteria.* Hoboken, NJ: Wiley.

[B70] Xu Z. Wu Y. Song L. Chinnathambi A. Ali Alharbi S. Fang L. (2020). Anticarcinogenic effect of zinc oxide nanoparticles synthesized from *Rhizoma paridis* saponins on Molt-4 leukemia cells. *J. King Saud Univ. Sci.* 32 1865–1871. 10.1016/j.jksus.2020.01.023

[B71] Yang D. Wang L. Wang T. Zhang Y. Zhang S. Luo Y. (2021). Plant growth-promoting rhizobacteria hn6 induced the change and reorganization of *Fusarium* microflora in the rhizosphere of banana seedlings to construct a healthy banana microflora. *Front. Microbiol.* 12:685408. 10.3389/fmicb.2021.685408 34354685 PMC8329250

[B72] Yi S. Li F. Wu C. Wei M. Tian J. Ge F. (2022). Synergistic leaching of heavy metal-polycyclic aromatic hydrocarbon in co-contaminated soil by hydroxamate siderophore: role of cation-pi and chelation. *J. Hazard. Mater.* 424:127514. 10.1016/j.jhazmat.2021.127514 34879514

[B73] Yun H. Lijian C. Wenhong Z. Yuhong D. Yongli W. Qiang W. (2007). Separation and identification of steroidal compounds with cytotoxic activity against human gastric cancer cell lines *in vitro* from the rhizomes of *Paris polyphylla* var. *chinensis*. *Chem. Nat. Compd.* 43 672–677. 10.1007/s10600-007-0225-8

[B74] Zahir Z. A. Shah M. K. Naveed M. Akhter M. J. (2010). Substrate-dependent auxin production by *Rhizobium phaseoli* improves the growth and yield of *Vigna radiata* L. under salt stress conditions. *J. Microbiol. Biotechnol.* 20 1288–1294. 10.4014/jmb.1002.02010 20890093

[B75] Zhanel G. G. Golden A. R. Zelenitsky S. Wiebe K. Lawrence C. K. Adam H. J. (2019). Cefiderocol: a siderophore cephalosporin with activity against carbapenem-resistant and multidrug-resistant gram-negative bacilli. *Drugs* 79 271–289. 10.1007/s40265-019-1055-2 30712199

[B76] Zhou H. Y. Han Y. Shi Q. Chen C. F. (2019). Directional transportation of a helic[6]arene along a nonsymmetric molecular axle. *J. Org. Chem.* 84 5872–5876 10.1021/acs.joc.9b00229 30900452

