# Supplementary material for: A Potential Biofertilizer—Siderophilic Bacteria Isolated From the Rhizosphere of Paris polyphylla var. yunnanensis
Source: Front Microbiol. 2022 May 9;13:870413. doi: 10.3389/fmicb.2022.870413 (PMC9125218; doi:10.3389/fmicb.2022.870413)
Supplement: Supplementary file 1 [file Data_Sheet_1.docx]

Supplementary Material

**Supplementary Table 1.** Accession numbers of 22 strains

| **Strains** | **Accession** |
| --- | --- |
| AS1 | MT291866.1 |
| AS2 | MW020341.1 |
| AS3 | MW020342.1 |
| AS4 | MW020343.1 |
| AS5 | MW025985.1 |
| AS6 | MW025986.1 |
| AS7 | MW025987.1 |
| AS8 | MT318666.1 |
| AS9 | MW025988.1 |
| AS10 | MT318656.1 |
| AS11 | MT318657.1 |
| AS12 | MT318658.1 |
| AS13 | MW025980.1 |
| AS14 | MW020304.1 |
| AS15 | MT318659.1 |
| AS16 | MT318660.1 |
| AS17 | MT318661.1 |
| AS18 | MT318662.1 |
| AS19 | MT318663.1 |
| AS20 | MT318664.1 |
| AS21 | MT318665.1 |
| AS22 | MW020339.1 |

**Supplementary Table 2.** Morphological observation of 22 isolates.

| **Number** | **Size** | **Shape** | **Edge** | **Transparency** | **Colour** |
| --- | --- | --- | --- | --- | --- |
| **AS1** | little | moist and round | regularity | opaque | yellow |
| **AS2** | big | moist, like nasal discharge | irregularity | transparent | colorless |
| **AS3** | big | moist and round, ruffles | regularity | translucent | white |
| **AS4** | big | moist, water droplets | regularity | translucent | white |
| **AS5** | big | moist, water droplets | regularity | translucent | white |
| **AS6** | big | moist, water droplets | regularity | translucent | white |
| **AS7** | big | moist, water droplets | regularity | translucent | white |
| **AS8** | little | drying and round, ruffles | regularity | opaque | yellow |
| **AS9** | big | moist, water droplets | regularity | translucent | white |
| **AS10** | little | drying and round, ruffles | regularity | opaque | yellow |
| **AS11** | little | drying and round, ruffles | regularity | opaque | yellow |
| **AS12** | little | drying and round, ruffles | regularity | opaque | yellow |
| **AS13** | little | drying and round, ruffles | regularity | opaque | yellow |
| **AS14** | big | mosit, like nasal discharge | irregularities | transparent | colorless |
| **AS15** | little | drying and round, ruffles | regularity | opaque | yellow |
| **AS16** | little | drying and round, ruffles | regularity | opaque | yellow |
| **AS17** | little | drying and round, ruffles | regularity | opaque | yellow |
| **AS18** | little | drying and round, ruffles | regularity | opaque | yellow |
| **AS19** | little | drying and round, ruffles | regularity | opaque | yellow |
| **AS20** | little | drying and round, ruffles | regularity | opaque | yellow |
| **AS21** | little | drying and round, ruffles | regularity | opaque | yellow |
| **AS22** | big | moist and round, ruffles | regularity | translucent | white |

**Supplementary Table 3.** Main physiological and biochemical characteristics of 22 bacterial strains.

| **Number** | **Starch hydrolysis** | **Gelatin liquefaction** | **Sugar fermentation** | **MR**  **test** | **Indole test** | **Voges-Proskauer test** | **Citrate test** | **Hydrogen sulfide** |
| --- | --- | --- | --- | --- | --- | --- | --- | --- |
| **AS1** | ＋ | + | - | － | － | + | - | ＋ |
| **AS2** | - | + | + | - | - | - | - | + |
| **AS3** | - | - | + | - | - | - | - | - |
| **AS4** | - | + | + | - | - | - | - | + |
| **AS5** | ＋ | － | ＋ | － | － | － | ＋ | - |
| **AS6** | ＋ | － | ＋ | － | － | + | ＋ | - |
| **AS7** | ＋ | － | ＋ | － | － | - | ＋ | - |
| **AS8** | － | + | － | － | － | - | - | － |
| **AS9** | + | － | ＋ | - | － | + | + | - |
| **AS10** | － | + | - | － | － | - | - | - |
| **AS11** | － | + | - | － | － | - | － | － |
| **AS12** | + | + | - | - | - | - | - | + |
| **AS13** | - | + | - | - | - | - | - | - |
| **AS14** | - | + | + | - | - | - | - | - |
| **AS15** | - | + | - | - | - | - | - | - |
| **AS16** | - | + | - | - | - | - | - | - |
| **AS17** | - | + | + | - | - | - | - | - |
| **AS18** | - | + | + | - | - | - | - | - |
| **AS19** | + | + | - | - | - | - | + | - |
| **AS20** | + | + | - | - | - | - | - | - |
| **AS21** | - | + | - | - | - | - | - | - |
| **AS22** | + | + | + | - | - | - | + | + |

Note: + means positive, - means negative


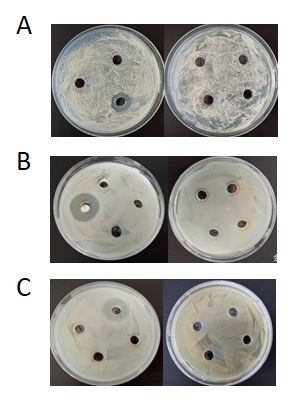


**Supplementary Figure 1.** The antimicrobial activity of cell-free supernatants from 22 isolates grown on LB. (**A**) Antimicrobial experiment with LB cell-free supernatant against *C. albicans*. N: LB medium negative control, P: Amphotericin positive control. (**B**) Antimicrobial experiment with LB cell-free supernatant against *Staphylococcus aureus*. N: LB medium negative control, P: cephalosporin positive control. (**C**) Antimicrobial experiment with LB cell-free supernatant against *Escherichia coli.* N: LB medium negative control, P: cephalosporin positive control. Represented by individual plate experiments, the data statistics are shown in Table 1.


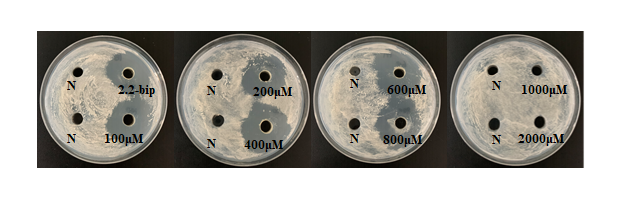


**Supplementary Figure 2.** Antibacterial activity detection with different concentrations of Fe^3+^. N, negative control. *Candida albicans* was cultured on PDA at 30°C for 14-16 h, and then spread plate cultivation on the PDA agar medium. Wells approximately 8 mm in diameter and 2 mm deep were made on the surface of the agar medium using a sterile borer. Each well was subsequently filled with 100 μL of test sample. The same amount of SA medium added with different amounts of iron was used as a negative control. The size of the bacteriostatic zone was observed after overnight culture.
